# Identifying Distinct Quality-of-Life Profiles in Prostate Cancer Patients: A Latent Profile Approach

**DOI:** 10.3390/curroncol33090532

**Published:** 2026-09-02

**Authors:** Linan Cheng

**Affiliations:** School of Nursing, Wenzhou Medical University, Wenzhou 325035, China; lncheng2024@wmu.edu.cn

**Keywords:** prostate cancer, quality of life, latent profile analysis, urinary dysfunction

## Abstract

**Background:** Prostate cancer substantially affects patients’ quality of life (QoL). However, whether distinct QoL profiles exist among patients remains unclear. **Objective:** This study aimed to identify latent QoL profiles among patients with prostate cancer and explore factors associated with profile membership. **Methods:** A cross-sectional study included 200 patients with prostate cancer recruited from a tertiary hospital in China between May and December 2024. QoL was assessed using the Functional Assessment of Cancer Therapy–Prostate (FACT-P). Latent profile analysis was performed using Mplus 8.3, and the optimal model was selected according to information criteria, entropy, and likelihood ratio tests. Multivariable logistic regression was used to examine factors associated with profile membership. **Results:** LPA identified two distinct subgroups: low QoL (32.5%) and high QoL (67.5%). Medium and heavy economic burden significantly increased odds of low QoL (OR = 4.13, 95% CI: 1.13–15.02, *p* = 0.032; OR = 11.12, 95% CI:1.55–79.86, *p* = 0.017). Urinary continence markedly reduced odds of low QoL (OR = 0.08, 95% CI: 0.02–0.25, *p* < 0.001). Longer diagnosis-to-treatment intervals were associated with membership in the low-QoL profile (1–3 months: OR = 2.99, 95% CI: 1.26–7.08, *p* = 0.013; >3 months: OR = 3.36, 95% CI: 1.02–11.07, *p* = 0.046). **Conclusions:** This study identified two QoL profiles among patients with prostate cancer. The findings suggest that person-centered QoL assessment may facilitate early identification of patients with greater supportive care needs and contribute to more individualized survivorship care.

## Simple Summary

Prostate cancer and its treatment can greatly affect patients’ quality of life, but not all patients experience the same challenges. Understanding these differences is important for providing more personalized care. In this study, we identified two distinct groups of patients with different levels of quality of life. Patients with greater financial burden, delayed treatment after diagnosis, and urinary incontinence were more likely to have poorer quality of life. These findings suggest that reducing financial stress, improving urinary care, and ensuring timely treatment may help improve the well-being of men living with prostate cancer. Our results provide practical evidence for healthcare professionals and policymakers to develop targeted supportive care strategies and improve survivorship outcomes.

## 1. Introduction

Improving quality of life (QoL) has become a central focus in cancer care, as patients face multifaceted physical, psychological, and social challenges that significantly impact long-term well-being [1,2]. Prostate cancer, one of the most prevalent malignancies among middle-aged and older men, contributes substantially to global male mortality [3]. Patients often experience treatment-related complications such as urinary incontinence, sexual dysfunction, and pelvic pain, which impose persistent health management burdens and directly affect treatment adherence and mental health [4]. Identifying heterogeneous QoL profiles and their determinants is therefore critical for developing tailored interventions to address these challenges.

QoL assessment has evolved into a key metric for evaluating holistic health outcomes, guiding clinical decision-making, and monitoring long-term survivorship [5]. Existing QoL assessment tools for prostate cancer patients include the European Organisation for Research and Treatment of Cancer Quality of Life Questionnaire-Core 30 (EORTC QLQ-C30) and the Functional Assessment of Cancer Therapy-General (FACT-G). The EORTC QLQ-C30 covers multiple dimensions, including physical functioning, role functioning, emotional functioning, cognitive functioning, social functioning, global health status/QoL, and a range of symptom scales such as fatigue, pain, nausea/vomiting, and others [6]. Similarly, the FACT-G evaluates four primary domains: physical well-being, social/family well-being, emotional well-being, and functional well-being [7]. Additionally, prostate cancer-specific modules like the EORTC QLQ-PR25 [8] and the FACT-Prostate (FACT-P) [9] extend these core instruments to assess disease-related symptoms and concerns, such as urinary, bowel, and sexual function. Together, these instruments demonstrate that QoL in prostate cancer is a multidimensional construct encompassing physical, emotional, social, functional, and disease-specific domains.

QoL represents a critical outcome in prostate cancer survivorship. The U.S. National Cancer Institute highlights that treatment-related side effects, including urinary and sexual dysfunction, are primary drivers of QoL decline in prostate cancer patients [10]. Moreover, QoL is influenced by a complex interplay of factors, such as treatment modality, social support, and financial burden [11]. These findings indicate that QoL is influenced by multiple clinical and socioeconomic factors rather than disease characteristics alone.

Despite these comprehensive measurement frameworks, analytic approaches in many empirical studies often reduce QoL to a total or average score, implicitly assuming that patients with similar overall scores have comparable experiences. Such approaches may overlook meaningful heterogeneity in patients’ overall QoL and patterns of functioning across multiple domains [12,13]. Clinical and psychosocial evidence suggests that prostate cancer patients differ considerably in their physical, emotional, social, functional, and disease-specific experiences, although the extent to which these differences reflect distinct multidimensional profiles or simply varying levels of overall QoL remains unclear [14,15].

Therefore, a person-centered analytical approach may be useful for exploring heterogeneity in QoL and identifying potential patient subgroups based on patterns across FACT-P domains. To address this gap, the present study used latent profile analysis (LPA) to identify subgroups of patients based on FACT-P domain scores and to examine factors associated with profile membership. We hypothesized that (1) patients could be classified into different QoL subgroups according to their FACT-P responses and (2) greater financial burden, poorer urinary status, and longer diagnosis-to-treatment intervals may be associated with membership in lower-QoL subgroups.

## 2. Methods

### 2.1. Study Design

This cross-sectional study was conducted among patients with prostate cancer to examine QoL profiles and their determinants.

### 2.2. Participants

From May to December 2024, a convenience sample of patients with histologically confirmed prostate cancer who had undergone surgical treatment was recruited from the urology department of a tertiary Grade A hospital in China. Inclusion criteria were as follows: (1) male patients aged ≥18 years; (2) histologically confirmed prostate cancer and having undergone surgical treatment; and (3) capacity to provide informed consent. Exclusion criteria comprised: (1) cognitive impairment or severe neuropsychological conditions affecting questionnaire completion; (2) active severe mental disorders (e.g., major depressive disorder); and (3) language barriers precluding comprehension.

The minimum sample size was initially estimated using the commonly recommended respondent-to-item ratio of 5–10 participants per questionnaire item for questionnaire-based studies [16]. With 39 questionnaire items, the target sample size ranged from 195 to 390 participants. After excluding invalid responses, 200 participants were included in the final analysis. Although no universally accepted sample size criterion exists for LPA, the present sample was considered adequate for exploratory LPA based on previous methodological recommendations [17,18].

### 2.3. Measures

A self-administered questionnaire was developed through literature review and expert consultation to collect information on age, place of residence, educational level, family relationship, average monthly family income (CNY), economic burden, time since diagnosis, age of onset, urinary status, tumor stage, diagnosis-to-treatment interval, treatment modality, and comorbidities. Economic burden was self-reported by participants and categorized as none, light, moderate, or heavy according to their perceived financial burden related to prostate cancer. Urinary status was categorized as no urinary incontinence, urinary incontinence, or urinary catheterization. Tumor stage was classified as clinical stages I–IV according to the medical records. Treatment modality was determined based on the treatment received before questionnaire administration and was categorized as radical prostatectomy alone or radical prostatectomy combined with chemotherapy.

The validated Simplified Chinese version of the Functional Assessment of Cancer Therapy–Prostate (FACT-P), originally developed by Esper et al., was used to assess QoL [19]. The 39-item instrument comprises the 27-item FACT-General (FACT-G), which assesses physical, social/family, emotional, and functional well-being, and the 12-item Prostate Cancer Subscale (PCS), which evaluates disease-specific symptoms. Items are rated on a 5-point Likert scale (0 = “Not at all” to 4 = “Very much”), with higher total scores indicating better QoL. In the present study, the scale showed excellent internal consistency (Cronbach’s α = 0.913).

### 2.4. Data Collection

The FACT-P was administered after surgery during the postoperative treatment period. Data were collected through face-to-face administration of structured paper questionnaires containing: (a) study information, (b) informed consent documents, (c) completion guidelines, and (d) the formal survey. Three trained researchers implemented rigorous protocols, including standardized participant briefing, real-time assistance using predefined scripts, immediate completeness verification and exclusion of questionnaires with more than 10% missing items or patterned responses, and dual independent data entry with cross-validation to ensure data quality, with all participants providing written informed consent and maintaining the right to withdraw without penalty. A total of 218 questionnaires were distributed and collected, among which 18 were excluded based on predefined validity criteria; thus, 200 valid questionnaires were retained for analysis. Because invalid questionnaires were removed at the data-cleaning stage and all retained questionnaires were complete, the final analytic dataset contained no missing values and no additional missing-data handling procedures were required.

### 2.5. Statistical Analysis

Data were analyzed using Microsoft Excel 2016 (Microsoft Corporation, Redmond, WA, USA) and IBM SPSS Statis-tics, version 25.0 (IBM Corp., Armonk, NY, USA). To ensure reproducibility, the analyses followed a sequential procedure. First, latent profile analysis (LPA) was conducted to identify unobserved QoL subgroups using the five FACT-P domains (physical well-being, social/family well-being, emotional well-being, functional well-being, and prostate cancer-specific concerns) as continuous indicators. To examine whether differences in domain score ranges influenced the latent profile solution, a sensitivity analysis was performed using standardized (z-score) FACT-P domain scores, and the results were compared with those of the primary analysis to evaluate the robustness of the identified profiles.

The optimal number of latent classes was determined using multiple model-fit indices, including the Akaike information criterion (AIC), Bayesian information criterion (BIC), sample-size adjusted BIC (aBIC), Lo–Mendell–Rubin likelihood ratio test (LMR), bootstrap likelihood ratio test (BLRT), and entropy. Lower AIC, BIC, and aBIC values, significant LMR and BLRT results (*p* < 0.05), and higher entropy values indicated better model fit. After class enumeration, differences in sociodemographic and clinical characteristics were compared using chi-square tests for categorical variables and one-way analysis of variance (ANOVA), as appropriate. Variables with *p* < 0.05 in the univariable analyses were entered into the multivariable logistic regression model to examine factors associated with QoL profile membership [17,20]. All statistical tests were two-sided, and *p* < 0.05 was considered statistically significant.

## 3. Results

### 3.1. Participant Characteristics

The study included 200 prostate cancer patients with a mean age of 68.44 ± 6.23 years. Demographic characteristics revealed: 43.5% aged 61–69 years; 53.0% urban residents; 41.0% with primary education or below; and 84.0% without religious affiliation. Regarding socioeconomic status: 51.5% reported moderate family relationships; 27.0% had monthly household incomes of ¥5000–10,000; and 71.0% experienced no financial burden. Clinical characteristics showed: 34.5% diagnosed at age 66–70; 49.0% with urinary incontinence; 67.0% at tumor stage II; 68.5% receiving treatment within 1 month of diagnosis; 84.5% of participants underwent radical prostatectomy alone, while 15.5% underwent radical prostatectomy combined with chemotherapy; and 41.0% with one comorbidity (see Table 1).

### 3.2. Latent Profile Analysis

**Figure 1:**
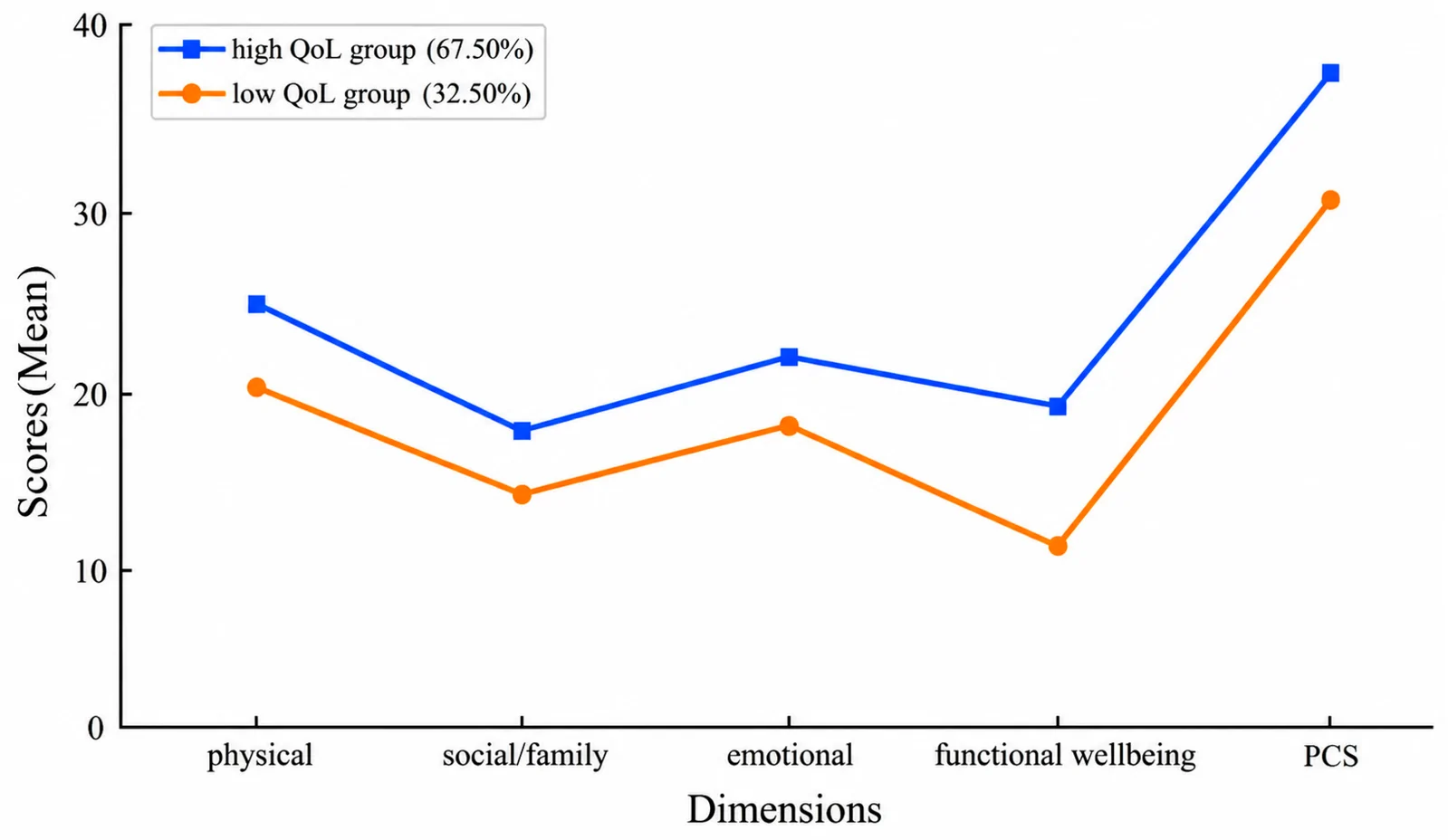
Two subgroups of QoL.

Model fit indices are presented in Table 2. LPA models with one to five latent profiles were estimated using Mplus 8.3. Although the AIC, BIC, and aBIC values decreased as the number of profiles increased, the three- and four-profile solutions were not retained because the LMR test was not significant (*p* > 0.05). The two-profile solution showed significant LMR and BLRT results (both *p* < 0.05), acceptable entropy (0.888), and class proportions of 67.5% and 32.5%; therefore, it was selected as the optimal model. A sensitivity analysis using standardized (z-score) FACT-P domain scores also supported the two-profile solution. Figure 1 presents the mean scores of the five FACT-P domains for the two latent profiles. Profile 1 had significantly higher scores across all domains and the total FACT-P score than Profile 2 (all *p* < 0.01). Accordingly, Profile 1 was designated as the high-QoL group and Profile 2 as the low-QoL group.

### 3.3. Factors Associated with Low QoL Profile Membership

The mean FACT-P score was 116.11 ± 15.37 among the 200 participants. Univariate analyses showed significant between-group differences in economic burden (χ^2^ = 17.597, *p* = 0.001), urinary status (χ^2^ = 27.449, *p* < 0.001), and diagnosis-to-treatment interval (χ^2^ = 6.747, *p* = 0.034) (Table 1).

Using the high-QoL profile as the reference group, multivariable logistic regression was performed to examine factors associated with membership in the low-QoL profile. Compared with patients reporting no financial burden, those with moderate and heavy financial burden had higher odds of belonging to the low-QoL profile (OR = 4.13, 95% CI: 1.13–15.02, *p* = 0.032; OR = 11.12, 95% CI: 1.55–79.86, *p* = 0.017, respectively). Patients without urinary incontinence had significantly lower odds of low-QoL profile membership than those requiring urinary catheterization (OR = 0.08, 95% CI: 0.02–0.25, *p* < 0.001). In addition, diagnosis-to-treatment intervals of 1–3 months and >3 months were associated with higher odds of low-QoL profile membership compared with treatment initiation within 1 month (OR = 2.99, 95% CI: 1.26–7.08, *p* = 0.013; OR = 3.36, 95% CI: 1.02–11.07, *p* = 0.046, respectively) (Table 3).

## 4. Discussion

This study aimed to identify latent QoL profiles among patients with prostate cancer using a person-centered analytical approach and to examine factors associated with profile membership. Two latent profiles were identified, primarily differing in overall QoL severity rather than in specific combinations of physical, social/family, emotional, functional, or prostate cancer-specific domains. These findings suggest that QoL heterogeneity in this population is better characterized by differences in overall health status than by domain-specific patterns. Accordingly, the two profiles are more appropriately interpreted as relatively low- and high-QoL groups rather than qualitatively distinct multidimensional QoL patterns. From a person-centered perspective, LPA provides a clinically interpretable description of patients who show consistently lower or higher QoL across multiple domains, which may help identify groups with differing supportive care needs. However, the present findings do not establish that profile-based classification provides additional clinical information beyond the continuous FACT-P total score. Further studies directly comparing LPA-based classification with continuous FACT-P scores are needed to determine whether LPA provides incremental clinical value.

The overall QoL observed in this study was generally comparable to previous reports in patients with prostate cancer, although slightly lower than that reported in some other populations [21,22]. Such differences are unlikely to be explained by disease characteristics alone and may instead reflect variations in socioeconomic conditions, healthcare accessibility, and the availability of supportive care across different settings [23,24,25]. As survival after prostate cancer continues to improve, patients increasingly face long-term physical, psychological, and social challenges that require ongoing multidisciplinary support [26,27,28]. Consequently, access to rehabilitation, symptom management, and psychosocial care may become as important as cancer treatment itself in determining long-term QoL [29,30,31]. Our findings therefore reinforce the importance of interpreting QoL within a broader biopsychosocial context and highlight the need to integrate supportive care into routine survivorship management.

Financial burden showed the strongest association with membership in the low-QoL profile, highlighting the important role of socioeconomic factors in prostate cancer survivorship. This finding is consistent with previous studies showing that economic hardship is associated with poorer health-related QoL across multiple cancer populations [32,33,34]. Financial constraints may influence QoL through several pathways, including reduced access to treatment and supportive care, lower treatment adherence, and increased psychological distress related to uncertainty and future financial security [35,36,37]. These challenges may collectively impair physical, emotional, and social functioning, resulting in poorer overall well-being. Although the present study assessed self-reported economic burden rather than financial toxicity, our findings further emphasize that financial difficulties should be routinely considered during survivorship assessment. Early identification of financially vulnerable patients may facilitate timely supportive interventions and contribute to more equitable, patient-centered cancer care.

Urinary dysfunction was another important factor associated with QoL. Patients requiring urinary catheterization or experiencing urinary incontinence were more likely to report poorer QoL than those with normal urinary function, consistent with previous studies in prostate cancer survivors [38,39,40]. Urinary problems can substantially affect daily functioning by limiting physical activity, disrupting social participation, and reducing patients’ confidence in maintaining normal routines. In addition to physical discomfort, concerns about urine leakage, catheter dependence, and altered body image may contribute to emotional distress and social withdrawal, thereby affecting multiple dimensions of QoL. These findings highlight that effective management of urinary symptoms should remain an integral component of survivorship care, with attention given to both functional recovery and psychosocial well-being. It should be noted that the prostate cancer-specific subscale of the FACT-P includes urinary symptom-related items. Therefore, some conceptual overlap between urinary status and the QoL outcome cannot be completely excluded and the observed association should be interpreted with appropriate caution.

Longer diagnosis-to-treatment intervals were associated with membership in the low-QoL profile. This finding is generally consistent with previous studies reporting that longer waiting periods are associated with poorer patient-reported outcomes and greater psychological distress [41,42,43]. However, in prostate cancer, treatment timing may also reflect appropriate clinical decision-making, including risk stratification and active surveillance, rather than harmful delay alone. Therefore, the observed association should not be interpreted as evidence of a causal relationship. Instead, these findings suggest that patients with longer diagnosis-to-treatment intervals may benefit from continued communication, psychological support, and routine QoL assessment throughout the treatment pathway.

The present findings have several clinical implications. First, routine QoL assessment should incorporate socioeconomic and treatment-related factors in addition to symptom severity, as these variables appear to contribute substantially to patients’ overall well-being. Second, the identification of a distinct low-QoL subgroup supports the use of person-centered approaches to identify patients who may benefit from more comprehensive supportive care. Finally, because financial burden, urinary dysfunction, and aspects of care delivery are potentially modifiable, multidisciplinary interventions targeting these factors may improve survivorship outcomes. Future longitudinal studies are needed to validate these findings and determine whether changes in these associated factors are accompanied by transitions between QoL profiles.

### Limitations

This study has several limitations. First, its cross-sectional design precludes causal inference. Second, several variables, including financial burden and urinary status, were self-reported and may therefore be subject to reporting bias. Third, participants were recruited from a single tertiary hospital, and the relatively small sample size may limit the generalizability of the findings and the ability to detect very small latent profiles. Fourth, although tumor stage and treatment modality were available, several clinically important variables, including metastatic status, treatment phase, surgical approach (e.g., open, laparoscopic, or robot-assisted surgery), treatment-related costs, catheter characteristics, and the interval between treatment and FACT-P assessment, were not collected, which may have limited the clinical interpretation of the identified QoL profiles. Although the sensitivity analysis using standardized FACT-P domain scores yielded the same two-profile solution, further analyses using more comprehensive data and alternative model specifications are needed to establish the robustness and reproducibility of the identified profiles. Finally, factors associated with latent profile membership were examined using conventional logistic regression rather than a three-step approach (e.g., R3STEP). Consequently, uncertainty in latent profile assignment was not explicitly incorporated into the regression analysis, and the resulting estimates should be interpreted with caution. In addition, because the FACT-P includes prostate cancer-specific urinary symptom items, some conceptual overlap between urinary status and the QoL outcome cannot be excluded. These methodological considerations should be taken into account when interpreting the findings. Future multicenter longitudinal studies with larger samples, more comprehensive clinical data, and analytical approaches that account for classification uncertainty are warranted to validate the identified QoL profiles and further examine factors associated with profile membership.

## 5. Conclusions

This study identified two distinct quality-of-life profiles among patients with prostate cancer using a person-centered analytical approach. Financial burden, urinary dysfunction, and longer diagnosis-to-treatment intervals were associated with membership in the low-QoL profile. These findings highlight the value of routine QoL assessment and person-centered risk stratification to identify patients who may benefit from more comprehensive supportive care. Future prospective studies are warranted to validate these findings and determine whether targeted interventions can improve long-term survivorship outcomes.

## Figures and Tables

**Table 1:** Univariate analysis of QoL among patients with prostate cancer (*n* = 200).

Variables		*n* (%)	High QoL (*n* = 135) *N* (%)	Low QoL (*n* = 65) *N* (%)	χ^2^	*p*
Age, years	≤60	25 (12.50)	17 (12.59)	8 (12.31)	0.049	0.976
	61–69	87 (43.50)	58 (42.96)	29 (44.62)		
	≥70	88 (44.00)	60 (44.44)	28 (43.08)		
Place of residence	City	106 (53.00)	75 (55.56)	31 (47.69)	1.089	0.364
	Rural	94 (47.00)	60 (44.44)	34 (52.31)		
Educational level	Illiteracy/Primary School	82 (41.00)	55 (40.74)	27 (41.54)	5.122	0.275
	Junior high school	60 (30.00)	42 (31.11)	18 (27.69)		
	High school/Technical secondary school	37 (18.50)	28 (20.74)	9 (13.85)		
	Junior college	13 (6.50)	6 (4.44)	7 (10.77)		
	University and above	8 (4.00)	4 (2.96)	4 (6.15)		
Religious belief	Yes	32 (16.00)	21 (15.56)	11 (16.92)	0.061	0.838
	No	168 (84.00)	114 (84.44)	54 (83.08)		
Family relationship	Good	81 (40.50)	59 (43.70)	22 (33.85)	5.179	0.075
	Mediocre	103 (51.50)	69 (51.11)	34 (52.31)		
	Bad	16 (8.00)	7 (5.19)	9 (13.85)		
Average monthly family income (CNY)	≤1000	17 (8.50)	9 (6.67)	8 (12.31)	3.692	0.595
	1000~3000	53 (26.50)	33 (24.44)	20 (30.77)		
	3000~5000	46 (23.00)	33 (24.44)	13 (20.00)		
	5000~10,000	54 (27.00)	38 (28.15)	16 (24.62)		
	10,000~30,000	24 (12.00)	17 (12.59)	7 (10.77)		
	≥30,000	6 (3.00)	5 (3.70)	1 (1.54)		
Economic burden	No	142 (71.00)	105 (77.78)	37 (56.92)	17.597	0.001
	Light	35 (17.50)	23 (17.04)	12 (18.46)		
	Medium	14 (7.00)	5 (3.70)	9 (13.85)		
	Heavy	9 (4.50)	2 (1.48)	7 (10.77)		
Age of onset	≤60	26 (13.00)	19 (14.07)	7 (10.77)	3.323	0.505
	61–65	26 (13.00)	14 (10.37)	12 (18.46)		
	66–70	69 (34.50)	49 (36.30)	20 (30.77)		
	71–75	58 (29.00)	40 (29.63)	18 (27.69)		
	≥76	21 (10.50)	13 (9.63)	8 (12.31)		
Urinary status	Urinary catheterization	33 (16.50)	17 (12.59)	16 (24.62)	27.449	<0.001
	Urinary incontinence	98 (49.00)	55 (40.74)	43 (66.15)		
	No urinary incontinence	69 (34.50)	63 (46.67)	6 (9.23)		
Clinical tumor stage	Stage I	28 (14.00)	18 (13.33)	10 (15.38)	1.945	0.584
	Stage II	134 (67.00)	88 (65.19)	46 (70.77)		
	Stage III	37 (18.50)	28 (20.74)	9 (13.85)		
	Stage IV	1 (0.50)	1 (0.74)	0 (0.00)		
Diagnosis-to-treatment interval	<1 month	137 (68.50)	100 (74.07)	37(56.92)	6.747	0.034
	1–3 months	46 (23.00)	27 (20.00)	19(29.23)		
	>3 months	17 (8.50)	8 (5.93)	9(13.85)		
Treatment modality	Radical prostatectomy alone	169 (84.50)	114 (84.44)	55(84.62)	0.001	1.000
	Radical prostatectomy combined with chemotherapy	31 (15.50)	21 (15.56)	10 (15.38)		
Number of chronic comorbidities	0	30 (15.00)	21 (15.56)	9 (13.85)		
	1	82 (41.00)	53 (39.26)	29 (44.62)	2.492	0.778
	2	41 (20.50)	30 (22.22)	11 (16.92)		
	3	28 (14.00)	17 (12.59)	11 (16.92)		
	4	14 (7.00)	11 (8.15)	3 (4.62)		
	5	5 (2.50)	3 (2.22)	2 (3.08)		

**Table 2:** Model fitting indexes for LPA in QoL.

Model	AIC	BIC	aBIC	LMR(P)	BLRT(P)	Entropy	Probabilities of Classes
One-profile	5461.731	5494.714	5463.033	—	—	—	—
Two-profile	5136.411	5189.184	5138.495	0.0004	<0.001	0.888	0.6750, 0.3250
Three-profile	5047.247	5119.810	5050.112	0.1016	<0.001	0.894	0.0642, 0.3358, 0.6000
Four-profile	5011.421	5103.774	5015.067	0.2739	<0.001	0.903	0.3365, 0.0623 0.0306, 0.5706
Five-profile	4974.600	5086.743	4979.027	0.3575	<0.001	0.868	0.0299, 0.1318, 0.0664, 0.3100, 0.4619

Note: AIC, Akaike information criterion; BIC, Bayesian information criterion; aBIC, sample-size-adjusted Bayesian information criterion; LMR-LRT, Lo–Mendell–Rubin adjusted likelihood ratio test; BLRT, bootstrap likelihood ratio test.

**Table 3:** Multivariable logistic regression analysis of factors associated with low-QoL profile membership (*N* = 200).

Variable	β	SE	OR	95%CI	*p*
Economic burden situation (ref. No)					
Light	0.044	0.439	1.045	0.442–2.467	0.921
Medium	1.418	0.659	4.127	1.134–15.021	0.032
Heavy	2.409	1.006	11.119	1.548–79.861	0.017
Urinary status (ref. Urinary catheterization)					
Urinary incontinence	−0.416	0.448	0.660	0.274–1.588	0.354
No urinary incontinence	−2.573	0.608	0.076	0.023–0.251	<0.001
Time from diagnosis to treatment reception (ref. <1 month)					
1–3 months	1.096	0.439	2.991	1.264–7.078	0.013
>3 months	1.213	0.608	3.362	1.021–11.071	0.046

Note: SE, standard error; OR, odds ratio; β, unstandardized coefficient; CI, confidence interval; *p* < 0.05.

## Data Availability

The datasets used and/or analyzed during the current study are available from the corresponding author on reasonable request.
